# Recommendations for Perioperative Care in Liver Resection: The EUPEMEN (EUropean PErioperative MEdical Networking) Protocol

**DOI:** 10.3390/medicina61060978

**Published:** 2025-05-26

**Authors:** Orestis Ioannidis, Aggeliki Koltsida, Elissavet Anestiadou, Jose M. Ramirez, Nicolò Fabbri, Javier Martínez Ubieto, Carlo Vittorio Feo, Antonio Pesce, Kristyna Rosetzka, Antonio Arroyo, Petr Kocián, Luis Sánchez-Guillén, Ana Pascual Bellosta, Adam Whitley, Alejandro Bona Enguita, Marta Teresa-Fernandéz, Stefanos Bitsianis, Savvas Symeonidis

**Affiliations:** 1 Fourth Department of Surgery, Medical School, Faculty of Health Sciences, Aristotle University of Thessaloniki, General Hospital “George Papanikolaou”, 57010 Thessaloniki, Greece; aggeliki.koltsida@gmail.com (A.K.); elissavetxatz@gmail.com (E.A.); sbitsiani@gmail.com (S.B.); simeonidissavvas@yahoo.com (S.S.); 2 Institute for Health Research Aragón, 50009 Zaragoza, Spain; jramirez@unizar.es (J.M.R.); jmte-zubieto@hotmail.com (J.M.U.); anapascual689@gmail.com (A.P.B.); secretariagerm@gmail.com (A.B.E.); mteresa@iisaragon.es (M.T.-F.); 3 Department of Surgery, Faculty of Medicine, University of Zaragoza, 50009 Zaragoza, Spain; 4 Department of Surgery, Azienda Unità Sanitaria Locale Ferrara, University of Ferrara, 44121 Ferrara, Italy; n.fabbri@ausl.fe.it (N.F.); cvfeo@unife.it (C.V.F.); antonio.pesce@ausl.fe.it (A.P.); 5 Department of Anesthesia, Resuscitation and Pain Therapy, Miguel Servet University Hospital, 50009 Zaragoza, Spain; 6 Department of Plastic Surgery, Second Faculty of Medicine, Charles University, Motol University Hospital, 150 06 Prague, Czech Republic; kristina.rosetzua@gmail.com; 7 Department of Surgery, Universidad Miguel Hernández Elche, Hospital General Universitario Elche, 03203 Elche, Spain; arroyocir@hotmail.com (A.A.); drsanchezguillen@gmail.com (L.S.-G.); 8 Grupo Español de Rehabilitación Multimodal (GERM), 50009 Zaragoza, Spain; 9 Department of Surgery, Second Faculty of Medicine, Charles University, Motol University Hospital, 150 06 Prague, Czech Republic; kocian.cz@gmail.com; 10 Department of Surgery, University Hospital Kralovske Vinohrady, 100 34 Prague, Czech Republic; whitley.adam@gmail.com

**Keywords:** liver resections, liver surgery, liver surgery guidelines, enhanced recovery after surgery, training and dissemination, perioperative care, EUPEMEN project

## Abstract

Hepatectomies play a crucial role in the multidisciplinary management of primary and secondary liver malignancies but are associated with significant risks, including 30-day mortality, morbidity, prolonged hospitalization, and increased resource utilization. Optimizing perioperative care remains a challenge; however, enhanced recovery programs have shown improved patient outcomes. The EUPEMEN (EUropean PErioperative MEdical Networking) protocol focuses on improving the perioperative management of liver resections through the establishment of interdisciplinary principles based on practical experience and theoretical frameworks from five European countries. This paper outlines the core elements of the EUPEMEN protocol, emphasizing strategies to minimize surgical stress, optimize perioperative care, and enhance postoperative recovery. The protocol is systematically designed to reduce postoperative mortality and morbidity, shorten hospital stays, and improve patient outcomes. The EUPEMEN guidelines address inconsistencies in surgical practice across Europe and are structured for implementation in various healthcare environments. “The protocol’s approach is designed to support improvements in perioperative care standards in liver resections and may serve as a practical and efficient tool for healthcare professionals, pending further clinical validation. The EUPEMEN protocol offers a standardized, evidence-based framework to enhance perioperative management in hepatectomies. By integrating multidisciplinary principles, the main target is to eliminate complications, improve surgical outcomes, and promote faster recovery. Its implementation across diverse clinical settings may contribute to advancing perioperative care standards for liver resections in Europe.

## 1. Introduction

A remarkable derangement in patients’ homeostasis and physiology is observed in major abdominal surgeries, including impaired pulmonary function, a catabolic state, elevated oxygen demand, persistent postoperative pain, nausea and vomiting, and impaired mobilization with a high possibility of venous thromboembolism, all of which could potentially contribute to patients’ prolonged hospitalization and recovery [1]. Fast-track surgery, or enhanced recovery after surgery (ERAS), was first conceptualized by Prof. Henrik Kehlet in the 1990s for colorectal surgery, resulting in dramatically improved surgical outcomes [2]. However, its universal application remains challenging due to variations in healthcare resources, professional practices, and cultural attitudes across Europe.

Across several abdominal operations, liver surgery is considered a complicated and major operation, entailing numerous inherent complications, such as intraoperative bleeding, hypotension and massive intravascular changes in fluid volume, prolonged operative time, and, postoperatively, coagulopathy, high bleeding risk, biliary leak, post-hepatectomy liver, and renal failure, as well as pulmonary complications. It should be highlighted that liver disease with cirrhosis is pre-existent in these patients, or they may have undergone chemotherapy, potentially leading to nodular regenerative hyperplasia and non-cirrhotic portal hypertension [3]. All the aforementioned conditions are very demanding for healthcare professionals, who are participating in these patients’ perioperative care. Hepatectomies are the cornerstone of the multidisciplinary approach to treating primary and secondary liver malignancies, with high risks of 30-day mortality and morbidity, which are disclosed to be at 14–55% [4] and 0–11.9% [5], respectively, and are correlated with resource utilization and prolonged hospitalization.

The chief objective of the study was the demonstration of the consensus-based methodology for perioperative patient care in liver resections. The protocol was created considering both expertise and clinical experience from a multidisciplinary team in several healthcare environments across Europe, and it provides customized suggestions than can be applied across various scenarios, serving the purpose of the standardization and enhancement of perioperative care in our daily clinical practice [6]. The EUPEMEN project, created using clinical insights and professional experience from four different European countries, has as its primary objective the training of younger colleagues in the distribution and accurate implementation of enhanced recovery frameworks in liver surgery.

## 2. The EUPEMEN Initiative

The EUropean PErioperative MEdical Networking (EUPEMEN) project’s goal has been to incorporate the combination of the clinical experience and the expertise of medical professionals who have a significant role in their countries in terms of delivering major change programs and to circulate the ERAS methodology in the European setting [7]. The team was designed in order to build up a homogenized educational ERAS manner that is able to be distributed through an online learning platform, in order to assist those who are engaged in applying evidence-based ERAS protocols in a specific approach. The ERASMUS + program (Agreement number 2020-1-ES01-KA203-082681) was supported by EUPEMEN.

The EUPEMEN initiative also contributes to global efforts aiming to refine perioperative care models by providing a replicable blueprint that can be adapted beyond Europe, especially in healthcare environments seeking to balance high clinical standards with local feasibility. The definitive goal of this manuscript is to analyze the fundamental points of the suggested guidelines for liver resections in the EUPEMEN protocol [8].

Based on their theoretical and clinical background and expertise, partners from five different university hospitals in Europe have collaborated with the fundamental aim of creating specific proposals for the highest standards of rehabilitation after surgery. “G. Papanikolaou—GPAP” General Hospital of Thessaloniki (Thessaloniki, Greece) with partners from Fundación Instituto de Investigación Sanitaria Aragón-IISA (Aragón, Spain), Universidad Miguel Hernández de Elche—UMH (Elche, Spain), Univerzita Karlova—CUNI (Nové Město, Czech Republic), and Azienda Unità Sanitaria Locale Ferrara—AUSLFE (Ferrara, Italy) have launched the project [9]. All the aforementioned institutions are well-known leaders in the surgical field and medical research, focusing on evidence-based protocols in order to enhance patient postoperative outcomes. The powerful consolidation of research into everyday clinical practice, in combination with the extensive experience in coordinating large-scale international projects, have significantly contributed to the development of the comprehensive, practical, and effective EUPEMEN protocol. It should be underlined that the target patient population includes adults and elderly individuals who have challenging medical histories, as well as diverse physiological responses to therapeutic management. In this setting, general surgeons with practical and theoretical experience in adult populations are the exclusive members of the protocol’s development team.

**Figure 1:**
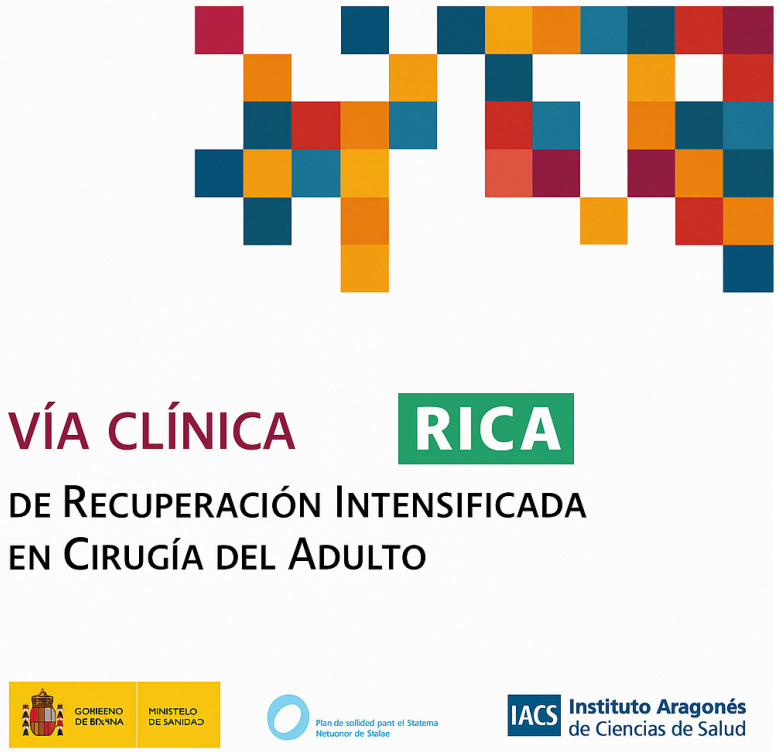
Cover page of the “Vía Clínica RICA” protocol document. This document outlines the *Clinical Pathway for Intensified Recovery in Adult Surgery (Recuperación Intensificada en Cirugía del Adulto—RICA)*. Developed by the Spanish Ministry of Health in collaboration with the Instituto Aragonés de Ciencias de la Salud (IACS) and the Red Española de Agencias de Evaluación, the protocol forms part of Spain’s national effort to standardize and enhance perioperative care and recovery. The colorful mosaic design symbolizes the interdisciplinary and integrated approach of the RICA pathway across various surgical specialties.

A non-systematic review of the existing international literature focusing on perioperative management in liver resections was performed by the European consensus panel. A multi-stage, structured procedure integrating a multidisciplinary experts’ team, including anesthesiologists, surgeons, and several other healthcare professionals employed in these organizations in Europe, was used. In 2015, based on the consensus of several scientific societies, the Clinical Pathway for Intensified Recovery in Abdominal Surgery (Via RICA) protocol for abdominal surgical procedures was initiated, highlighting the purpose of strengthening postoperative recovery and improving patient outcomes and safety (Figure 1). Six years later, the standardization of the principles of surgical care was performed by the renovation of the Via RICA protocol in compliance with recovery protocols incorporated in several surgical fields [10].

The target groups of the EUPEMEN project are divided into direct and indirect. The first category includes medical professionals contiguously participating in surgical patients’ care, such as surgeons, anesthesiologists, nurses, and those who are related to interdisciplinary care, like gastroenterologists, radiotherapists, nutritionists, physiotherapists, pathologists, and oncologists. The second category consists of health center administrators, quality coordinators, and clinical managers, who will exploit the enhanced recovery program in terms of the reduction of hospitalization’s duration and other resources’ optimization. Finally, patients and their supporting network, patients’ associations, and the project’s stakeholders reap benefits from the implementation of the protocol in surgical practice.

**Figure 2:**
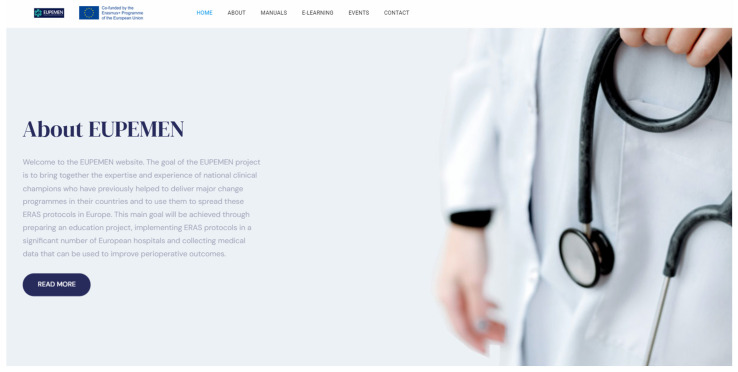
Overview page of the open-access EUPEMEN website, outlining the project’s mission to implement ERAS protocols across Europe through education, collaboration, and outcome-based improvements.

**Figure 3:**
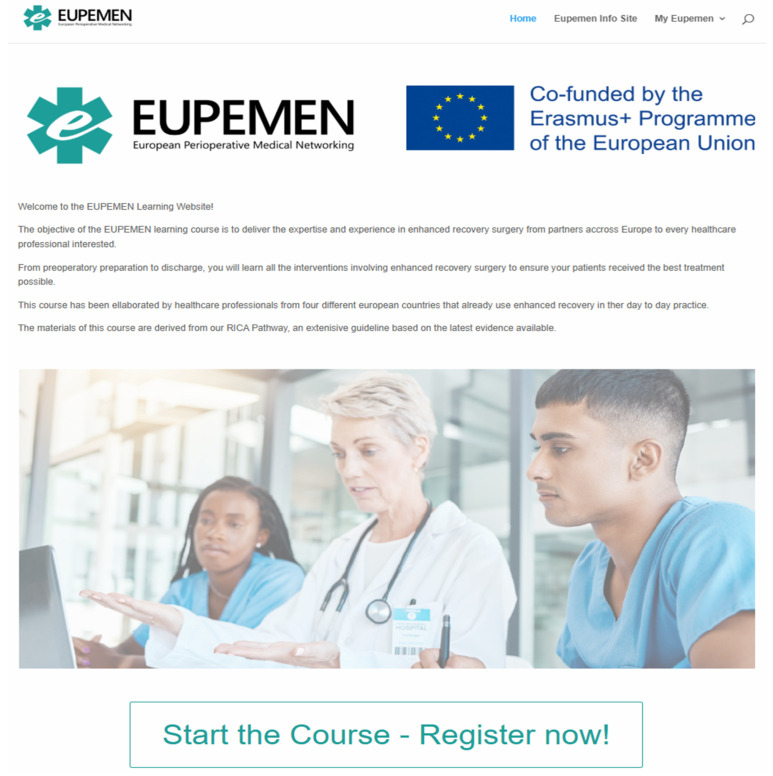
Homepage of the open-access EUPEMEN learning platform, offering enhanced recovery training based on the RICA Pathway, co-funded by the Erasmus + Programme.

The technical activities of the EUPEMEN project were essential in the structured development and dissemination of enhanced perioperative care protocols across Europe. Central to these efforts was the preparation of the EUPEMEN Multimodal Rehabilitation manual, which included six procedure-specific modules: Bariatric Surgery, Oesophageal Surgery, Gastric Cancer Surgery, Colon Surgery, Hepatobiliary Surgery, and Urgent Abdominal Surgery, including appendectomy and small bowel obstruction. To facilitate training and collaboration, the EUPEMEN online platform (https://eupemen-learning.com, accessed on 24 April 2023) was developed, offering an e-learning course and a collaborative space for protocol development and engagement (Figure 2 and Figure 3). A “train-the-trainer” initiative was implemented to equip local educators with the skills necessary to teach and promote the protocol in their respective hospitals. Dissemination activities included five Multiplier events—one in each partner country—to promote protocol awareness and adoption, alongside four transnational meetings to support strategic coordination. Additionally, the Spanish Via RICA protocol was translated into English, broadening accessibility. The project culminated in the EUPEMEN Protocols Training Programme, which trained 200 multidisciplinary professionals and facilitated implementation in at least five European hospitals. These efforts also led to the creation of a professional network capable of supporting long-term adoption, auditing practices, and ensuring quality implementation. The expected long-term outcomes include reduced postoperative complications, shortened hospital stays, enhanced recovery, and overall cost savings for healthcare systems.

## 3. The EUPEMEN Protocol in Liver Surgery

**Figure 4:**
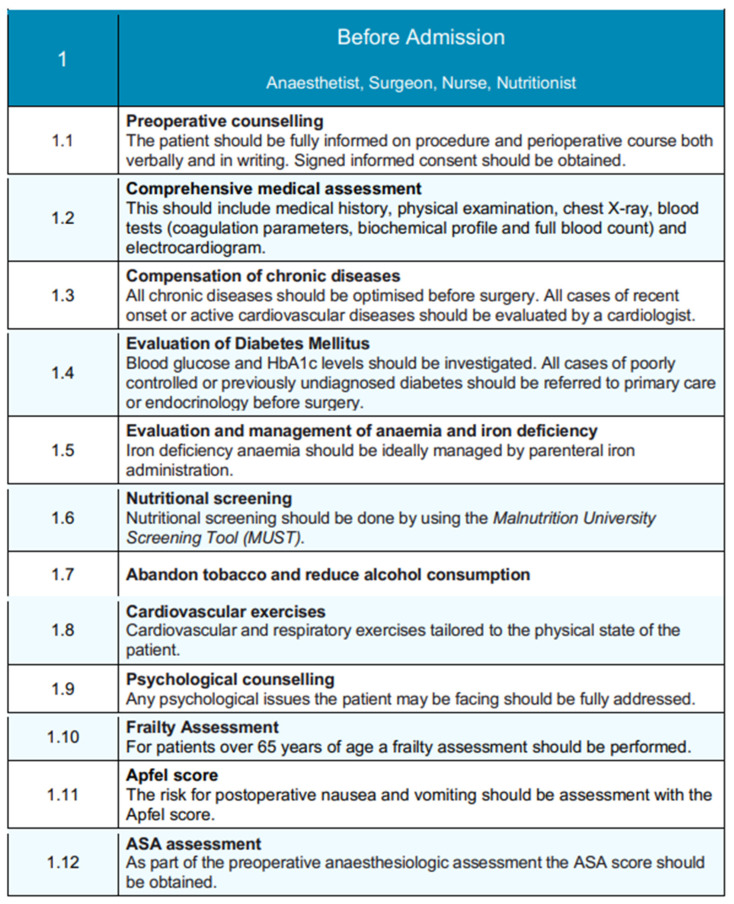
Pre-admission phase of the EUPEMEN protocol, detailing multidisciplinary preoperative assessments and optimization steps involving the anesthetist, surgeon, nurse, and nutritionist.

**Figure 5:**
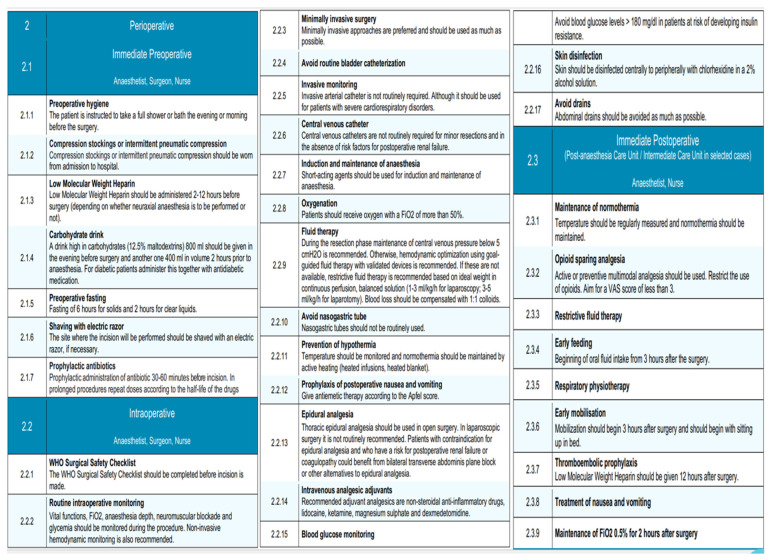
Perioperative phase of the EUPEMEN protocol, including immediate preoperative, intraoperative, and immediate postoperative interventions led by the surgical, anesthetic, and nursing teams to enhance recovery after liver surgery.

**Figure 6:**
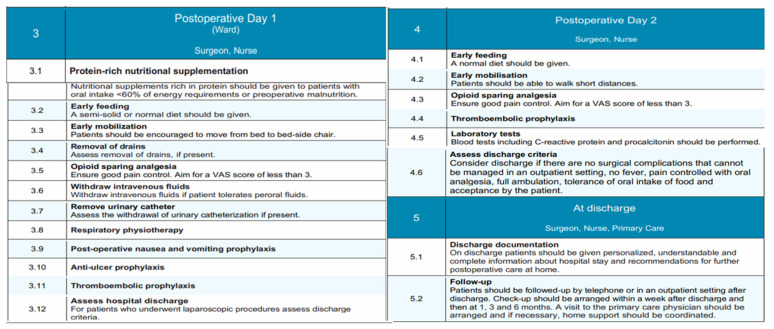
Postoperative and discharge phases of the EUPEMEN protocol, outlining nutritional support, mobilization, analgesia, thromboembolic prophylaxis, discharge planning, and follow-up to ensure enhanced recovery after liver surgery.

The structure of the EUPEMEN protocol for liver resections includes five different and discrete stages: (1) a phase before the patient’s admission, involving four healthcare professionals, an anesthetist, a surgeon, a nurse, and a nutritionist (Figure 4); (2) the perioperative stage, including the stage inside the operation room and the immediate postoperative, as well as a (probable) stay in the intensive care unit (ICU, where an anesthetist, a surgeon, and a nurse are involved; and finally (3) the postoperative phase, which lasts until the completion of the second postoperative day (POD2), the remaining hospitalization, when appropriate, and discharge of the patient. An expanded, phase-by-phase visual presentation of the EUPEMEN protocol follows (Figure 4, Figure 5 and Figure 6).

### 3.1. Before Admission

The preoperative stage of the patient planning to undergo a liver resection is performed—even before admission—by the collaborative work of four healthcare professionals: an anesthetist, a surgeon, a nurse, and a nutritionist.

The first step involves preoperative counseling, where patients are thoroughly informed about the procedure, the perioperative stage, and the potential complications both verbally and in writing [11], signing the informed consent [12,13].

Following that, the comprehensive medical assessment of the patient begins with the medical record, clinical examination, electrocardiogram, chest X-ray, and the laboratory tests, such as full blood count, biochemical profile, and coagulation parameters. Chronic conditions should be optimized prior to surgery, and, especially, a cardiologist must evaluate all of the cases of recent onset or active cardiovascular diseases [14]. Diabetes Mellitus, anemia, and iron deficiency have to be investigated and controlled by referring to endocrinologists before surgery or by parenteral iron administration, respectively. Furthermore, nutritional screening should be obtained by using the Malnutrition University Screening Tool (MUST) [15]. In terms of the patient’s habits, tobacco abandonment [16] and alcohol consumption cessation must be ensured prior to surgery [17], while cardiovascular and respiratory exercises tailored to the physical state of the patient have been proven beneficial. In this setting, any psychological issues should be fully addressed. Last, when it comes to patients aged over 65 years, a frailty assessment is crucial [18], as well as the calculation of the Apfel Score for the evaluation of the likelihood for postoperative nausea and vomiting (PONV) [19]. Anesthesiologists are recommended to calculate preoperatively the ASA score in all the patients prepared for a liver resection.

### 3.2. Perioperative Phase

#### 3.2.1. Immediate Preoperative

The immediate preoperative stage of the EUPEMEN principles in liver surgery is mostly performed by a surgeon, an anesthetist, and a nurse. All of them should ensure the patient’s preoperative hygiene, with a bath in the evening or a few hours prior to the operation. When it comes to the thromboembolic prophylaxis, two important measures should be taken prior to the operation: intermittent pneumatic compression or compression stockings and the administration of low molecular weight heparin (LMWH). More specifically, the first must be worn at hospital admission, whereas the second has to be administered 2–12 h prior to surgery, depending on whether neuraxial anesthesia is to be performed or not [20,21].

Furthermore, patients are encouraged to consume 800 mL of a high-carbohydrate drink (12.5% maltodextrin) the night prior to surgery together with a second dose of 400 mL in volume a couple of hours prior to anesthesia [22,23]. Especially for diabetic patients, this has to be administered together with their antidiabetic medication. Pre-operatively, refraining from solid food for 6 h and for clear liquids for around 2 h is mandatory [24].

Perioperative care bundles to avert surgical site infections (SSIs) are greatly recommended, while antibiotic chemoprophylaxis has to be administered, according to the local policy of the department, 30–60 min before the incision in all cases [25,26]. Last but not least, it is important that the incision site be shaved with an electric razor, in case it is deemed necessary.

#### 3.2.2. Intraoperative Phase

In this phase of liver resection, the execution of the World Health Organization (WHO) Surgical Safety Checklist is mandatory, because it contributes to the safety of the patient [27,28]. In this phase, the teamwork of the surgeon, the anesthesiologist, and the nurse holds a significant role [8].

In accordance with the guidelines of the EUPEMEN protocol, anesthesiologists are accountable for the rapid sequence of induction (RSI) for anesthesia using short-acting agents, in combination with no face mask ventilation, which is considered the predominant technique for eliminating the risk of aspiration [29]. Its maintenance, along with the maintenance of routine intraoperative monitoring, is considered crucial. This stage entails non-invasive hemodynamic monitoring, including vital functions and the evaluation of blood pressure; electrocardiography monitoring by an electrocardiogram with five leads, with V5 and DII highly suggested [30]; the continuous calculation of the fraction of inspired oxygen (FiO_2_), since oxygen should be administered with a FiO_2_ of more than 50% [31]; pulse oximetry for oxygen saturation; and capnography (EtCO_2_) [32]. Invasive monitoring with the use of arterial catheters is not routinely required; however, it should be considered for patients with severe cardiorespiratory disorders. Central venous catheters are not routinely mandatory for minor liver resections and when risk factors for postoperative renal failure are absent. Furthermore, anesthesiologists should monitor the core body temperature continuously and active heating with the combined usage of heated blankets and heated infusions is essential for normothermia maintenance [33]. Goal-directed fluid therapy (GDT) is a key point, targeting the perioperative hemodynamic optimization of the patient and, consequently, the minimization of the associated complication rate [34]. In this setting, the implementation of non-invasive hemodynamic monitoring devices could be recommended. However, in case they are absent, restrictive fluid therapy in continuous perfusion, based on the ideal weight and the proposed surgical approach, is advised. More specifically, this counts for 1–3 mL/kg/h for laparoscopic and 3–5 mL/kg/h for open resections. Blood loss should be compensated with 1:1 colloids [35,36]. The evaluation of PONV risk with the use of Apfel score and the relevant administration of antiemetic therapy is also indicated. Nasogastric tubes should not be regularly used. Relating to the intraoperative analgesic adjuvants, non-steroidal anti-inflammatory drugs, lidocaine, ketamine, magnesium sulphate, and dexmedetomidine are typically preferable. When it comes to the benefits of epidural analgesia, the thoracic one should be used in open liver resections, whereas it is not commonly mentioned in laparoscopic procedures. In patients with contraindication for epidural analgesia and who may present with high probability for coagulopathy or postoperative renal failure, bilateral transverse abdominis plane block or other alternatives could be proved valuable [37].

Regarding the surgical team’s responsibilities during this stage of the EUPEMEN protocol, they are encouraged to incline towards minimally invasive procedures as much as possible, considering their previous professional experience, in combination with the hospital’s resources and abilities. Urinary catheterization and abdominal drains should be used only when required. Lastly, perioperative care bundles to intercept and reduce SSIs are encouraged, while peripheral skin disinfection with a 2% alcohol solution of chlor-hexidine is considered one of the first priorities in the operation room [38].

Finally, according to the multidisciplinary care of the patients, the responsible medical team ought to attain glycemic control perioperatively. Depending on the protocols of each local hospital for diabetics undergoing surgery, patients with a risk of developing insulin resistance (elderly and obese patients) should not present with glucose levels higher than 180 mg/dL in their blood and, particularly, in surgeries which last more than sixty minutes [39].

#### 3.2.3. Immediate Postoperative Phase

The EUPEMEN protocol’s immediate postoperative stage for liver resections takes place in the Post-anesthesia Care Unit or, in selected cases, in the Intermediate Care Unit and is predominantly conducted by an anesthetist and a nurse. In order to prevent hypothermia and hyposaturation, their crucial priorities are the maintenance of normothermia, as well as the routine oxygen saturation measurement for FiO_2_ 0.5% maintenance for 2 h postoperatively [40]. In addition, opioid-sparing multimodal active or preventive analgesia is considered a significant element of this stage [41], aiming for a VAS score of less than 3. Restrictive fluid therapy is highly recommended, since it has been correlated with fewer complications, such as decreased rates of infectious cardiovascular and respiratory complications [41]. Extended prophylaxis for thromboembolic events using low-molecular-weight heparin, compression stockings, or intermittent compression must be administered as specified by the local hospital policy and usually 12 h after the operation. Patients’ mobilization should be encouraged to begin 3 h after surgery, together with oral fluid intake and respiratory physiotherapy [42,43].

### 3.3. Postoperative Day 1 and 2 (Ward)

This stage represents a significant part of enhanced recovery protocols, in which the collaboration between the patient, the surgeon, and the nurse is essential for the patient’s quick rehabilitation and uneventful postoperative course. At present, the surgical ward is where the perioperative care takes place. During this period, early feeding with a semi-solid or normal diet, along with protein-rich nutritional supplementation, should be emphasized, precisely to patients with oral intake <60% or energy requirements or preoperative malnutrition [44]. Additionally, early mobilization from bed to bed-side chair and full ambulation [45], in combination with respiratory physiotherapy with a breathing device, has proven beneficial [46,47]. Sufficient opioid-sparing analgesia should be ensured, along with adequate post-operative nausea, vomiting, and anti-ulcer prophylaxis. Of course, the early assessment of drains and urinary catheter withdrawal, if present, should be taken under consideration. In this stage, laboratory tests including C-reactive protein and procalcitonin must be performed. The continuous administration of intravenous fluids is recommended for the first days for those who have insufficient oral fluid intake. For those who can tolerate peroral fluid administration, the cessation of intravenous infusions must be followed. Patients after liver resections might be susceptible to thromboembolic events, and this should be highlighted postoperatively. In this setting, thromboembolic prophylaxis with compression stockings, the administration of LMWH, and intermittent pneumatic compression devices have to be preserved in the postoperative period, in conformity with the hospital’s policy.

### 3.4. Discharge

Even from the first postoperative day, patients, and especially those who underwent laparoscopic procedures, must be assessed for the completion of early discharge criteria and home-readiness. In more detail, the responsible healthcare professionals have to consider discharge if surgical complications are absent and patients are afebrile, can control pain with oral analgesic medication, are capable of full ambulation, and tolerant of oral intake of food and, of course, if they accept it. At this stage, which remains an essential stage of perioperative care, an understandable, personalized, and comprehensive report on the hospital stay, as well as advice for home, should be given to all patients [48]. They should continue thromboembolic prophylaxis for 28 days after surgery [49]. In terms of their follow up, they must be invited in the first seven days afterwards to the outpatient clinic or contacted by phone. Additional follow-up visits should be organized for 1, 3, and 6 months after discharge and in compliance with the local hospital policy; patients’ support at their home with a primary care physician has to be coordinated [50].

## 4. Discussion

Optimizing postoperative outcomes requires implementing empirical patients’ care concepts consolidated into a multimodal rehabilitation protocol [2]. The background experience with the well-known “fast-track” methodology on colorectal surgery has produced Enhanced Recovery After Surgery (ERAS), a methodology developed in 1997 by several surgeons based in Northern Europe and initiated by Henrik Kehlet [51,52]. The fundamental point of this protocol was to minimize organisms’ response to surgical stress by enhancing the perioperative nutritional status, encouraging early postoperative feeding as well as opioid-sparing analgesia [53]. Among its benefits, ERAS has been proved to eliminate complications during the postoperative phase, as well as the length of stay (LoS), after different types of surgery [54,55]. Over time, multiple colleagues from all over the world and with different surgical backgrounds have reported noteworthy advancements in terms of the time and quality of recovery after several operations, leading to the creation of “ERAS Society” in 2010 in Sweden, as a global non-profit medical academic society [56]. Five years later, in an additional endeavor to improve postoperative results, a thorough enhanced recovery protocol with instructions for abdominal surgery, called the Intensified Recovery in Abdominal Surgery (Via RICA) protocol, came into existence and was published by the Ministry of Health, Social Services, and Equality and the Aragon Health Sciences Institute [10]. ERAS guidelines for liver resections were published for the first time in 2016 6, and, since then, several meta-analyses and publications in the international literature have demonstrated that the implementation of this methodology in liver surgery was responsible for the reduction of postoperative complication rate, LoS, and costs [57,58,59].

It is particularly essential to highlight the importance of compliance to all ERAS items. However, this encounters several challenges and barriers, and its international implementation and adoption are uncertain. Interestingly, the inadequate application of these principles has driven a lower quality of healthcare services and poorer post-operative outcomes [60]. In order to fill this gap, the EUPEMEN project used data extracted by the aforementioned RICA protocol. It is important to mention that Via RICA represents a thorough systematic review of the already known theoretical background on this topic, which evaluated and ranked in detail the evidence correlated to every domain of surgical practice, guaranteeing that all these concepts in the EUPEMEN project have been based in the highest extent of the accessible evidence. This procedure has been thoroughly developed in the Via RICA protocol. The EUPEMEN project for liver resection builds on this foundation, emphasizing the clinical implementation, as well as the tailored adjustments of the recommendations.

## 5. Enhanced Recovery After Surgery (ERAS) for Liver Resections

The Enhanced Recovery After Surgery (ERAS) program, originally developed for colorectal surgery, has since expanded to various surgical disciplines with proven benefits in reducing postoperative complications, shortening hospital stays, and optimizing recovery [61]. These protocols have been lately applied in hepato-pancreatico-biliary surgery (HPB), promoting conceivable benefits in perioperative morbidity and mortality [6].

ERAS guidelines for liver resections were first published in 2016, and since then, numerous publications have demonstrated that their implementation leads to the elimination of postoperative complications, costs, and LoS, as well as to the improvement of postoperative outcomes. ERAS protocol entails 25 recommendations regarding the preoperative, intraoperative and postoperative phases of liver resections. More especially, nine of them had a high level of evidence, and three novel items in the preoperative stage were introduced for the first time, highlighting the paramount importance of adherence to all of the suggestions. Patients with cirrhosis undergoing liver resections could be safely managed with the ERAS protocol, although further data would be beneficial for this category [62].

Interestingly, several difficulties in the adoption of ERAS guidelines have been observed, including: multidisciplinary cooperation, cultural and organizational obstacles, resistance to change, regularization, resource allocation, patient coaching, and monitoring. In this setting, the current publication analyzes a methodology for combined effort and teamwork among numerous hospitals in Europe to develop guidelines and platforms for their distribution, especially for liver resections.

Although the EUPEMEN protocol is firmly grounded in the ERAS philosophy, it brings several novel elements that extend beyond standard ERAS recommendations. The EUPEMEN project directly addresses these challenges by providing a harmonized, European-centric approach. Developed through an international collaboration involving university hospitals from Spain, Italy, the Czech Republic, and Greece, EUPEMEN integrates multidisciplinary expertise to create procedure-specific protocols tailored to the complexities of local healthcare settings [63]. For liver surgery, the protocol is not a simple adaptation of ERAS principles but a structured framework that incorporates the experience of existing multimodal pathways, such as Via RICA, and includes precise recommendations for preoperative assessment, intraoperative strategies, and postoperative recovery phases.

A distinctive feature of EUPEMEN is its comprehensive implementation strategy. Beyond protocol design, the project has developed open-access educational resources, multilingual rehabilitation manuals, and an online learning platform to facilitate widespread adoption. Importantly, the ‘train-the-trainer’ model ensures that local healthcare teams receive tailored education, enabling effective and sustainable implementation even in varied resource settings [64]. Regular auditing and quality improvement initiatives further ensure that adherence to the protocol is monitored and continuously improved.

Moreover, the EUPEMEN protocol emphasizes the role of patient-centered care and multidisciplinary involvement, extending participation beyond core surgical teams to include dietitians, physiotherapists, psychologists, and primary care providers. This holistic approach not only enhances recovery outcomes but also fosters communication among healthcare professionals, addressing a well-documented gap in ERAS implementation.

Overall, EUPEMEN represents an evolution of the ERAS framework, specifically designed to overcome barriers to uniform perioperative care in Europe. By offering a practical, evidence-informed, and widely accessible protocol, EUPEMEN aims to support improvements in surgical outcomes, a standardization of perioperative care, and resource utilization across diverse European settings, pending future clinical validation. Table 1 summarizes the key differences between EUPEMEN and the standard ERAS protocol.

The rationale for developing a European-specific protocol such as EUPEMEN stems from the diverse healthcare landscapes found across Europe. Unlike ERAS, which provides broadly applicable guidelines, EUPEMEN has been specifically designed to address variations in healthcare systems, clinical practices, and resource availability that exist between European countries [7]. The differences in hospital infrastructure, perioperative pathways, reimbursement systems, and professional cultures often limit the effective and uniform application of generic protocols. For instance, certain components of ERAS may be difficult to implement in centers with fewer resources or in regions where multidisciplinary coordination is less established. EUPEMEN responds to these challenges by offering structured, adaptable recommendations that are sensitive to the practical realities of healthcare delivery in Europe [8]. Moreover, Europe presents unique demographic challenges that justify a tailored approach. With an ageing population and a higher prevalence of multimorbidity in surgical patients, especially in countries with older demographic profiles, there is an increased need for comprehensive perioperative optimization. EUPEMEN addresses this by integrating specific considerations for frailty, comorbidities, and patient-centered counseling, ensuring that recommendations are relevant not only to high-volume university hospitals but also to smaller regional centers. By leveraging the experience of multiple expert centers across Europe, the protocol harmonizes best practices while respecting local constraints, thus enhancing clinical applicability and patient safety across diverse healthcare settings. Finally, the collaborative nature of EUPEMEN promotes sustainable adoption throughout Europe. By combining educational initiatives, a multilingual online platform, and the ‘train-the-trainer’ strategy, the protocol facilitates widespread dissemination and effective uptake, regardless of country-specific barriers. Regular auditing mechanisms ensure continuous improvement and adherence, fostering a culture of quality and shared learning among European institutions [9]. In this way, EUPEMEN not only builds upon the foundations of ERAS but also addresses its limitations, offering a pragmatic and regionally relevant solution for advancing perioperative care in Europe.

## 6. Conclusions

In conclusion, the EUPEMEN protocol provides a standardized, evidence-based framework for optimizing perioperative care in liver resections. By integrating multi-disciplinary principles and best practices, it aims to reduce postoperative morbidity and mortality, improve patient recovery, and shorten hospital stays. The structured approach of EUPEMEN enhances surgical outcomes by minimizing variability in perioperative management across different healthcare settings in Europe. Furthermore, its implementation is intended to streamline resource utilization and promote a more efficient and patient-centered approach to hepatectomy care, although its effectiveness remains to be evaluated in clinical settings. Continued collaboration, training, and dissemination of the protocol will be essential for its successful adoption and long-term impact on surgical care.

## Figures and Tables

**Table 1:** EUPEMEN vs. standard ERAS protocol: Structured enhancement of perioperative care in Europe.

Aspect	Standard ERAS Protocol	EUPEMEN Protocol
Scope of Application	Global recommendations, generalized pathways	European-focused, procedure-specific adaptation
Development Process	Expert consensus by international societies	Multidisciplinary European network, consensus + clinical input
Procedure Specificity	General principles, adaptable	Highly detailed, procedure-specific protocols (e.g., liver, gastric, appendicitis)
Implementation Tools	Guidelines and clinical pathways	Online platform, multilingual manuals, train-the-trainer, audits
Target Audience	Surgeons, anesthetists, perioperative teams	Full multidisciplinary teams, incl. dietitians, nurses, primary care
Barriers Addressed	Limited guidance on barriers	Cultural, organizational, and resource barriers actively addressed
Monitoring and Sustainability	Variable adherence, limited audits	Embedded auditing and continuous improvement processes
Patient Engagement	Emphasized but center-dependent	Standardized counseling, shared decision-making, informed consent
Aim	Enhance recovery and reduce hospital stay	Harmonize European care, improve outcomes, reduce costs

## Data Availability

The data presented in this study are available at https://eupemen-learning.com, (accessed on 1 November 2024).

## Abbreviations

The following abbreviations are used in this manuscript:

EUPEMEN	European Perioperative Medical Networking
ERAS	Enhanced Recovery After Surgery
Via RICA	Via Clinica de Recuperacion Intensificada en Cirugia Del Adulto
MUST	Malnutrition Universal Screening Tool
PONV	Postoperative Nausea and Vomiting
ASA	American Society of Anesthesiologists
LMWH	Low Molecular Weight Heparin
SSIs	Surgical Site Infections
WHO	World Health Organization
RSI	Rapid Sequence Induction
FiO2	Fraction of Inspired Oxygen
EtCO2	End-tidal carbon dioxide
GDT	Goal-directed fluid therapy
VAS	Visual Analogue Scale
LoS	Length of Stay

## References

[1] Agarwal V., Divatia J.V. (2019). Enhanced recovery after surgery in liver resection: Current concepts and controversies. Korean J. Anesthesiol..

[2] Kehlet H., Wilmore D.W. (2008). Evidence-based surgical care and the evolution of fast-track surgery. Ann. Surg..

[3] Bayramov N., Mammadova S. (2022). A review of the current ERAS guidelines for liver resection, liver transplantation and pancreatoduodenectomy. Ann. Med. Surg..

[4] Damania R., Cocieru A. (2017). Impact of enhanced recovery after surgery protocols on postoperative morbidity and mortality in patients undergoing routine hepatectomy: Review of the current evidence. Ann. Transl. Med..

[5] van Dam R.M., Hendry P.O., Coolsen M.M.E., Bemelmans M.H.A., Lassen K., Revhaug A., Fearon K.C.H., Garden O.J., Dejong C.H.C. (2008). Initial experience with a multimodal enhanced recovery programme in patients undergoing liver resection. Br. J. Surg..

[6] Melloul E., Hübner M., Scott M., Snowden C., Prentis J., Dejong C.H.C., Garden O.J., Farges O., Kokudo N., Vauthey J. (2016). Guidelines for Perioperative Care for Liver Surgery: Enhanced Recovery After Surgery (ERAS) Society Recommendations. World J. Surg..

[7] Ioannidis O., Anestiadou E., Ramirez J.M., Fabbri N., Ubieto J.M., Feo C.V., Pesce A., Rosetzka K., Arroyo A., Kocián P. (2024). The EUPEMEN (EUropean PErioperative MEdical Networking) Protocol for Acute Appendicitis: Recommendations for Perioperative Care. J. Clin. Med..

[8] Pesce A.M.P.F., Ramírez J.M., Fabbri N., Ubieto J.M., Bellosta A.P., Arroyo A., Sánchez-Guillén L., Whitley A., Kocián P., Rosetzka K. (2024). The EUropean PErioperative MEdical Networking (EUPEMEN) project and recommendations for perioperative care in colorectal surgery: A quality improvement study. Int. J. Surg..

[9] Ioannidis O., Ramirez J.M., Ubieto J.M., Feo C.V., Arroyo A., Kocián P., Sánchez-Guillén L., Bellosta A.P., Whitley A., Enguita A.B. (2023). The EUPEMEN (EUropean PErioperative MEdical Networking) Protocol for Bowel Obstruction: Recommendations for Perioperative Care. J. Clin. Med..

[10] Ramirez J.M., Ruiz-López P., Gurumeta A.A. CLINICAL PATHWAY Recovery Intensification for Optimal Care in Adult’s Surgery. https://www.researchgate.net/publication/354723117.

[11] Broadbent E., Kahokehr A., Booth R.J., Thomas J., Windsor J.A., Buchanan C.M., Wheeler B.R., Sammour T., Hill A.G. (2012). A brief relaxation intervention reduces stress and improves surgical wound healing response: A randomised trial. Brain Behav. Immun..

[12] Kruzik N. (2009). Benefits of Preoperative Education for Adult Elective Surgery Patients. AORN J..

[13] Ronco M., Iona L., Fabbro C., Bulfone G., Palese A. (2012). Patient education outcomes in surgery: A systematic review from 2004 to 2010. Int. J. Evid.-Based Healthc..

[14] Kristensen S.D., Knuuti J., Saraste A., Anker S.D., Bøtker H.E., De Hert S., Ford I., Gonzalez-Juanatey J.R., Gorenek B., Heyndrickx G.R. (2014). 2014 ESC/ESA Guidelines on non-cardiac surgery: Cardiovascular assessment and management: The Joint Task Force on non-cardiac surgery: Cardiovascular assessment and management of the European Society of Cardiology (ESC) and the European Society of Anaesthesiology (ESA). Eur. Heart J..

[15] Deftereos I., Djordjevic A., Carter V.M., McNamara J., Yeung J.M., Kiss N. (2021). Malnutrition screening tools in gastrointestinal cancer: A systematic review of concurrent validity. Surg. Oncol..

[16] Gaskill C.E., Kling C.E., Varghese T.K., Veenstra D.L., Thirlby R.C., Flum D.R., Alfonso-Cristancho R. (2017). Financial benefit of a smoking cessation program prior to elective colorectal surgery. J. Surg. Res..

[17] Shabanzadeh D.M., Sørensen L.T. (2015). Alcohol consumption increases post-operative infection but not mortality: A systematic review and meta-analysis. Surg. Infect..

[18] Dalton A., Zafirova Z. (2018). Preoperative Management of the Geriatric Patient: Frailty and Cognitive Impairment Assessment. Anesthesiol. Clin..

[19] Apfel C.C., Greim C.A., Haubitz I., Grundt D., Goepfert C., Sefrin P., Roewer N. (1998). The discriminating power of a risk score for postoperative vomiting in adults undergoing various types of surgery. Acta Anaesthesiol. Scand..

[20] Afshari A., Fenger-Eriksen C., Monreal M., Verhamme P. (2018). European guidelines on perioperative venous thromboembolism prophylaxis. Eur. J. Anaesthesiol..

[21] Vivas D., Roldán I., Ferrandis R., Marin F., Roldan V., Tello-Montoliu A., Ruiz-Nodar J.M., Gómez-Doblas J.J., Martín A., Llau J.V. (2018). Perioperative and Periprocedural Management of Antithrombotic Therapy: Consensus Document of SEC, SEDAR, SEACV, SECTCV, AEC, SECPRE, SEPD, SEGO, SEHH, SETH, SEMERGEN, SEMFYC, SEMG, SEMICYUC, SEMI, SEMES, SEPAR, SENEC, SEO, SEPA, SERVEI, SECOT and AEU. Rev. Española De Cardiol. (Engl. Ed.).

[22] Bilku D.K., Dennison A.R., Hall T.C., Metcalfe M.S., Garcea G. (2014). Role of preoperative carbohydrate loading: A systematic review. Ann. R. Coll. Surg. Engl..

[23] Md S., Mccall J., Gp H., Soop M., Nygren J. (2014). Preoperative Carbohydrate Treatment for Enhancing Recovery after Elective Surgery (Review). http://www.thecochranelibrary.com.

[24] Brady M.C., Kinn S., Stuart P., Ness V. (2003). Preoperative fasting for adults to prevent perioperative complications. Cochrane Database Syst. Rev..

[25] Allegranzi B., Zayed B., Bischoff P., Kubilay N.Z., de Jonge S., de Vries F., Gomes S.M., Gans S., Wallert E.D., Wu X. (2016). New WHO recommendations on intraoperative and postoperative measures for surgical site infection prevention: An evidence-based global perspective. Lancet Infect. Dis..

[26] Badia J.M., Casey A.L., Petrosillo N., Hudson P.M., Mitchell S.A., Crosby C. (2017). Impact of surgical site infection on healthcare costs and patient outcomes: A systematic review in six European countries. J. Hosp. Infect..

[27] De Jager E., McKenna C., Bartlett L., Gunnarsson R., Ho Y.H. (2016). Postoperative Adverse Events Inconsistently Improved by the World Health Organization Surgical Safety Checklist: A Systematic Literature Review of 25 Studies. World J. Surg..

[28] Haynes A.B., Weiser T.G., Berry W.R., Lipsitz S.R., Breizat A.-H.S., Dellinger E.P., Herbosa T., Joseph S., Kibatala P.L., Lapitan M.C.M. (2009). A Surgical Safety Checklist to Reduce Morbidity and Mortality in a Global Population. N. Engl. J. Med..

[29] Wallace C., McGuire B. (2014). Rapid sequence induction: Its place in modern anaesthesia. Contin. Educ. Anaesth. Crit. Care Pain.

[30] Pahade A., Mowar A., Singh V., Kharayat U. (2022). Intraoperative Electrocardiogram Monitoring Induced Bispectral Index Interference—A Misleading Heart–Mind Connection. J. Indian Coll. Anaesthesiol..

[31] Becker D.E., Casabianca A.B. (2009). Respiratory Monitoring: Physiological and Technical Considerations. Anesth. Prog..

[32] Tarello D., Giogà F., Lauterio A., Becchetti C., Perricone G., Santi G., Ragazzi M., Monti G., Lazzeri M. (2024). Respiratory and physical therapy in the intensive care unit after liver transplantation for acute-on-chronic liver failure: A case report. Monaldi Arch. Chest Dis..

[33] Koenen M., Passey M., Rolfe M. (2017). “Keeping Them Warm”—A Randomized Controlled Trial of Two Passive Perioperative Warming Methods. J. Perianesthesia Nurs..

[34] Liu H., Kendrick J., Kaye A., Tong Y., Belani K., Urman R., Hoffman C. (2019). Goal-directed fluid therapy in the perioperative setting. J. Anaesthesiol. Clin. Pharmacol..

[35] Makaryus R., Miller T.E., Gan T.J. (2018). Current concepts of fluid management in enhanced recovery pathways. Br. J. Anaesth..

[36] Bacchin M.R., Ceria C.M., Giannone S., Ghisi D., Stagni G., Greggi T., Bonarelli S. (2016). Goal-directed fluid therapy based on stroke volume variation in patients undergoing major spine surgery in the prone position. Spine.

[37] Anyaehie K.B., Duryea E., Wang J., Echebelem C., Macias D., Sunna M., Ogunkua O., Joshi G.P., Gasanova I. (2022). Multimodal opioid-sparing pain management for emergent cesarean delivery under general anesthesia: A quality improvement project. BMC Anesthesiol..

[38] Dean H.F., King E., Gane D., Hocking D., Rogers J., Pullyblank A. (2020). Introduction of a care bundle effectively and sustainably reduces patient-reported surgical site infection in patients undergoing colorectal surgery. J. Hosp. Infect..

[39] Joshi G.P., Chung F., Vann M.A., Ahmad S., Gan T.J., Goulson D.T., Merrill D.G., Twersky R. (2010). Society for ambulatory anesthesia consensus statement on perioperative blood glucose management in diabetic patients undergoing ambulatory surgery. Anesth. Analg..

[40] Xu R., Hu X., Sun Z., Zhu X., Tang Y. (2023). Incidence of postoperative hypothermia and shivering and risk factors in patients undergoing malignant tumor surgery: A retrospective study. BMC Anesthesiol..

[41] Ghai B., Jafra A., Bhatia N., Chanana N., Bansal D., Mehta V. (2022). Opioid sparing strategies for perioperative pain management other than regional anaesthesia: A narrative review. J. Anaesthesiol. Clin. Pharmacol..

[42] Castelino T., Fiore J.F., Niculiseanu P., Landry T., Augustin B., Feldman L.S. (2016). The effect of early mobilization protocols on postoperative outcomes following abdominal and thoracic surgery: A systematic review. Surgery.

[43] de Almeida E., de Almeida J., Landoni G., Galas F.R.B.G., Fukushima J., Fominskiy E., de Brito C., Cavichio L., de Almeida L., Ribeiro-Jr U. (2017). Early mobilization programme improves functional capacity after major abdominal cancer surgery: A randomized controlled trial. Br. J. Anaesth..

[44] Sun Y., Yang Z., Tan H. (2014). Perioperative nutritional support and fluid therapy in patients with liver diseases. Hepatobiliary Surg. Nutr..

[45] Wolk S., Linke S., Bogner A., Sturm D., Meißner T., Müssle B., Rahbari N.N., Distler M., Weitz J., Welsch T. (2019). Use of Activity Tracking in Major Visceral Surgery—The Enhanced Perioperative Mobilization Trial: A Randomized Controlled Trial. J. Gastrointest. Surg..

[46] Kendall F., Oliveira J., Peleteiro B., Pinho P., Bastos P.T. (2018). Inspiratory muscle training is effective to reduce postoperative pulmonary complications and length of hospital stay: A systematic review and meta-analysis. Disabil. Rehabil..

[47] Alaparthi G.K., Augustine A.J., Anand R., Mahale A. (2016). Comparison of Diaphragmatic Breathing Exercise, Volume and Flow Incentive Spirometry, on Diaphragm Excursion and Pulmonary Function in Patients Undergoing Laparoscopic Surgery: A Randomized Controlled Trial. Minim. Invasive Surg..

[48] Shepperd S., Lannin N.A., Clemson L.M., Mccluskey A., Cameron I.D., Barras S.L. (2013). Discharge planning from hospital to home. Cochrane Database Syst. Rev..

[49] Golemi I., Salazar Adum J.P., Tafur A., Caprini J. (2019). Venous thromboembolism prophylaxis using the Caprini score. Dis.-A-Mon..

[50] Pieper B., Sieggreen M., Freeland B., Kulwicki P., Frattaroli M., Sidor D., Stasik S., Salerno C., Weissfeld L., Bindas E. (2006). Wound Care: Discharge Information Needs of Patients After Surgery. J. Wound Ostomy Cont. Nurs..

[51] Kehlet H. (1997). Multimodal approach to control postoperative pathophysiology and rehabilitation. Br. J. Anaesth..

[52] Taurchini M., Del Naja C., Tancredi A. (2018). Enhanced Recovery After Surgery: A patient centered process. J. Vis. Surg..

[53] Senturk J.C., Kristo G., Gold J., Bleday R., Whang E. (2017). The Development of Enhanced Recovery after Surgery Across Surgical Specialties. J. Laparoendosc. Adv. Surg. Tech..

[54] Coolsen M.M.E., Van Dam R.M., Van Der Wilt A.A., Slim K., Lassen K., Dejong C.H.C. (2013). Systematic review and meta-analysis of enhanced recovery after pancreatic surgery with particular emphasis on pancreaticoduodenectomies. World J. Surg..

[55] Greco M., Capretti G., Beretta L., Gemma M., Pecorelli N., Braga M. (2014). Enhanced recovery program in colorectal surgery: A meta-analysis of randomized controlled trials. World J. Surg..

[56] Ljungqvist O., Young-Fadok T., Demartines N. (2017). The History of Enhanced Recovery after Surgery and the ERAS Society. J. Laparoendosc. Adv. Surg. Tech..

[57] Li L., Chen J., Liu Z., Li Q., Shi Y. (2017). Enhanced recovery program versus traditional care after hepatectomy: A meta-analysis. Medicine.

[58] Zhao Y., Qin H., Wu Y., Xiang B. (2017). Enhanced recovery after surgery program reduces length of hospital stay and complications in liver resection. Medicine.

[59] Noba L., Rodgers S., Chandler C., Balfour A., Hariharan D., Yip V.S. (2020). Enhanced Recovery After Surgery (ERAS) Reduces Hospital Costs and Improve Clinical Outcomes in Liver Surgery: A Systematic Review and Meta-Analysis. J. Gastrointest. Surg..

[60] Martin D., Roulin D., Grass F., Addor V., Ljungqvist O., Demartines N., Hübner M. (2018). A multicentre qualitative study assessing implementation of an Enhanced Recovery After Surgery program. Clin. Nutr..

[61] Mithany R.H., Daniel N., Shahid M.H., Aslam S., Abdelmaseeh M., Gerges F., Gill M.U., Abdallah S.B., Hannan A., Saeed M.T. (2023). Revolutionizing Surgical Care: The Power of Enhanced Recovery After Surgery (ERAS). Cureus.

[62] Joliat G., Kobayashi K., Hasegawa K., Thomson J., Padbury R., Scott M., Brustia R., Scatton O., Cao H.S.T., Vauthey J. (2023). Guidelines for Perioperative Care for Liver Surgery: Enhanced Recovery After Surgery (ERAS) Society Recommendations 2022. World J. Surg..

[63] Ioannidis O., Anestiadou E., Ramirez J.M., Fabbri N., Ubieto J.M., Feo C.V., Pesce A., Rosetzka K., Arroyo A., Kocián P. (2025). Improving Perioperative Care in Gastric Surgery: Insights from the EUropean PErioperative MEdical Networking (EUPEMEN) Project. J. Clin. Med..

[64] Hollis J.L., Seward K., Kocanda L., Collins C.E., Tully B., Brett K., Hunter M., Foureur M., Schumacher T., Lawrence W. (2022). Evaluating a train-the-trainer model for scaling-up Healthy Conversation Skills training: A pre-post survey using the Theoretical Domains Framework. Patient Educ. Couns..

